# *Dracocephalum moldavica L*. Extracts Protect H9c2 Cardiomyocytes against H_2_O_2_-Induced Apoptosis and Oxidative Stress

**DOI:** 10.1155/2020/8379358

**Published:** 2020-05-02

**Authors:** Min Jin, Hui Yu, Xia Jin, Lailai Yan, Jingyu Wang, Zhanli Wang

**Affiliations:** ^1^Department of Laboratorial Science and Technology, School of Public Health, Peking University, Beijing 100191, China; ^2^School of Public Health, Baotou Medical College, Baotou 014060, China; ^3^Inner Mongolia Key Laboratory of Disease-Related Biomarkers, The Second Affiliated Hospital, Baotou Medical College, Baotou 014030, China; ^4^Inner Mongolia Autonomous Region People's Hospital, Hohhot 010010, China; ^5^Vaccine Research Center, School of Public Health, Peking University, Beijing 100191, China; ^6^Peking University Medical and Health Analysis Center, Peking University, Beijing 100191, China

## Abstract

**Materials and Methods:**

The petroleum ether (petrol), dichloromethane (CH_2_Cl_2_), ethyl acetate (EtOAc), and n-butyl alcohol (n-BuOH) fractions were isolated from alcohol extracts of *D*. *moldavica L*. Total phenolic and flavonoid contents and *in vitro* antioxidant activities of different fractions were evaluated. H9c2 cells were then treated with *D*. *moldavica L*. extracts before challenging with H_2_O_2_. Cell viability was determined by colorimetric assay, and ELISA was used to measure the levels of lactate dehydrogenase (LDH), malondialdehyde (MDA), and superoxide dismutase (SOD). Apoptosis levels and mitochondrial membrane potential were measured by flow cytometry. The expressions of cell apoptosis regulatory proteins caspase-3, Bax, and Bcl-2 were determined by western blotting.

**Results:**

Our results demonstrated that the EtOAc fraction from *D. moldavica L*. ethanol extract, which is rich in phenolic and flavonoid active constituents, had the strongest free radical scavenging activity. Additionally, this fraction increased H_2_O_2_-induced reduction in cell viability, SOD activity, and mitochondrial membrane potential. It also reduced H_2_O_2_-induced elevation in ROS production, contents of LDH and MDA, and H9c2 apoptosis. We further found that the EtOAc fraction increased Bcl-2 expression, while it decreased caspase-3 and Bax expressions induced by H_2_O_2_ in H9c2 cells.

**Conclusions:**

Our data revealed that the EtOAc fraction from *D. moldavica L*. ethanol extract ameliorates H_2_O_2_-induced cardiotoxicity via antiapoptotic and antioxidant mechanisms.

## 1. Introduction

Ischemic heart disease (IHD), the most common cause of death worldwide, is a chronic disease leading to myocardial ischemia, hypoxia, and necrosis [1]. The mechanisms involved in IHD are complicated. Accumulating evidence has revealed that oxidative stress plays an important role in the pathogenesis of myocardial ischemia-reperfusion (I/R) injury [2]. Oxidative stress occurs as a consequence of a redox imbalance in cells between prooxidants and antioxidants [3]. The overproduction of reactive oxygen species (ROS) and impairment of endogenous antioxidant systems may lead to the release of inflammatory cytokines and the enhancement of reactive aldehydes such as malondialdehyde (MDA) [4]. The increase in oxidative stress may also result in mitochondrial dysfunction, eventually leading to the activation of cardiac apoptotic signaling, further exacerbating ischemic injury [5]. The exogenous administration of antioxidants or the upregulation of endogenous antioxidants is an important approach for the prevention of I/R-induced cardiac injury [6]. Therefore, a pharmacological agent with antioxidant properties might be an effective strategy for cardioprotection. Plants often contain several potential active components which possess antioxidant properties such as phenols, flavonoids, terpenoids, and saponins [7]. Recently, several studies have revealed that herbal extracts containing antioxidant compounds exert a cardioprotective effect [8, 9].

*Dracocephalum moldavica L.* is a common perennial species that is widely used as a folk medicine for the treatment of cardiovascular diseases [10]. Several studies have shown that the extracts of *D. moldavica L.* possess high antioxidant activity [11, 12]. Previous studies also confirmed that the total flavonoid extract of *D. moldavica L.* can efficiently attenuate ischemia-induced myocardial injury, and its mechanism may be related to the improvement of myocardial oxidative stress states and regulation of the antiapoptotic signaling pathways [13, 14]. *D. moldavica L*. has been reported to contain flavonoids, glycoside, tannins, steroid, saponins, and phenolic compounds [15]. However, the antioxidant fraction and antioxidative stress mechanism of the alcohol extracts of *D*. *moldavica L*. have not been comprehensively described. This work is aimed at identifying the antioxidant fraction of *D. moldavica L.* extracts and at further investigating its antioxidant stress effects. Moreover, the cardioprotective effects of *D. moldavica L.* component in H9c2 cardiomyocytes under H_2_O_2_-induced oxidative stress and its underlying mechanisms were also investigated.

## 2. Materials and Methods

### 2.1. Chemicals and Reagents

2,2-Diphenyl-1-picrylhydrazylradical (DPPH), dimethyl sulfoxide (DMSO), 2,2′-azinobis (3-ethylbenzothiazoline-6-sulfonicacid) diammonium salt (ABTS), potassium persulfate, and hydrogen peroxide (H_2_O_2_) were purchased from Sigma-Aldrich (St. Louis, MO, USA). The cell culture products were purchased from Thermo Fisher Scientific Inc. (Waltham, MA, USA). The kits used to determine the MDA content and lactate dehydrogenase (LDH) and superoxide dismutase (SOD) activities were obtained from Jiancheng Bioengineering Institute (Nanjing, China). The fluorescent dye 5,5′,6,6′-tetrachloro-1,1′,3,3′-tetraethylbenzimidazolyl-carbocyanine iodide (JC-1) was purchased from Sigma-Aldrich (St. Louis, MO, USA), and the annexin V/propidium iodide (PI) apoptosis detection kit was obtained from Invitrogen (Eugene, OR, USA). General laboratory reagents were purchased from Sinopharm Chemical Reagent Co., Ltd. (Beijing, China). Antibodies of anti-Bax and anti-cleaved-caspase-3 were purchased from Millipore (Beverly, MA, USA), and antibodies of anti-Bcl-2, anti-pro-caspase-3, and anti-*β*-actin were purchased from Sangon Biotech (Shanghai, China). All chemical reagents were at least of analytical grade.

### 2.2. Plant Material and Extract Preparation

The aerial parts of *D*. *moldavica L.* were collected from the county of Tongliao, Inner Mongolia, China. The plant materials were air-dried and ground into a powder. The plant powder and the solvent (1 : 10 *v*/*v*) were placed in the flask of a constant-speed blender with a digital display (60 W, China) for reflux extraction twice with the following conditions: ethanol and water (65 : 35 *v*/*v*) as the solvent, 60°C as the extraction temperature, and 120 min as the extraction time. Subsequently, the extracts were concentrated in a rotary evaporator (Yarong RE-2000A, China) under reduced pressure for ethanol removal. The obtained ethanol extracts were then separated with petroleum ether (petrol), dichloromethane (CH_2_Cl_2_), ethyl acetate (EtOAc), and n-butyl alcohol (n-BuOH) using separating funnels. The resultant fractions were concentrated, dried, and stored at -20°C for further analyses.

### 2.3. Quantification of the Total Phenolic and Flavonoid Contents

The total phenolic content of each fraction was determined using the Folin-Ciocalteu method [16], whereas the total flavonoid content was measured using a spectrophotometric method as described previously [16]. Moreover, high-performance liquid chromatography (HPLC) was used for the qualitative analysis of the EtOAc fraction of *D. moldavica L*. ethanol extract with the standards (tallianine, rosmarinic acid, luteolin, apigenin, and diosmetin) as the references as described previously [17].

### 2.4. *In Vitro* Antioxidant Activity

The DPPH radical and ABTS radical scavenging activity was measured using the method as described previously [18]. Briefly, different dilutions of each fraction of *D. moldavica L*. ethanol extract were prepared. An aliquot of each dilution was mixed vigorously with a methanol solution of DPPH or with a water solution of ABTS. The absorbance was measured at 517 nm or 734 nm. Methanol or water was used as a blank control. The superoxide anion radical scavenging activity and the hydroxyl radical scavenging activity of each fraction were determined using a commercially available kit obtained from Jiancheng Bioengineering Institute (Nanjing, China).

### 2.5. Cell Culture and Treatment

The embryonic rat heart-derived H9c2 cell line was obtained from the Bank of the Chinese Academy of Sciences (Shanghai, China). Cells were cultivated in DMEM with 10% FBS and 100 mg/mL penicillin/streptomycin under atmospheric conditions of 95% air/5% CO_2_ at 37°C. H9c2 cells were then divided into three groups: control, model, and EtOAc fraction groups. The EtOAc fraction groups were pretreated with various concentrations of *D. moldavica L*. extracts (0.01, 0.05, 0.1, 0.3, 0.5, and 1.0 *μ*g/mL) for 24 h. No extracts were exposed to the control group and the model group. The model group and the EtOAc fraction groups were then exposed to H_2_O_2_ (150 mM) for 4 h, and the control group was treated with DMEM medium.

### 2.6. Cell Viability

Cell viability was determined using the Cell Counting Kit-8 (DOJINDO, Tokyo, Japan) according to the manufacturer's instructions. The absorbance of the culture medium in each well was recorded at 450 nm using a microplate reader (Bio-Rad, Hercules, CA, USA) to determine the cell viability. All experiments were repeated at least three times.

### 2.7. Measurement of MDA, LDH, and SOD Levels

The cultured supernatant and cells were collected after different treatments to determine the LDH and SOD activities as well as the MDA level using the corresponding commercially available kits obtained from Jiancheng Bioengineering Institute (Nanjing, China) following the manufacturer's instructions.

### 2.8. Determination of Intracellular ROS

The intracellular ROS levels in the H9c2 cells were measured using the fluorescent probe, 2′,7′dichlorodihydrofluorescein diacetate (DCFH-DA, Sigma-Aldrich, St. Louis, MO, USA). The cells were stained with 10 mM DCFH-DA for 20 min in the dark at 37°C. Then, fluorescence was measured using a fluorometer (U-RFLT50, OLYMPUS, Japan) at an excitation wavelength of 485 nm and an emission wavelength of 530 nm.

### 2.9. Detection of Apoptosis

Apoptosis was measured by flow cytometry using an annexin V-FITC apoptosis detection kit (Sigma-Aldrich, St. Louis, MO, USA) according to the manufacturer's instructions. Cells were incubated with 5 *μ*L of FITC-annexin V and 1 *μ*L of PI working solution (100 *μ*g/mL) for 15 min in the dark at room temperature. Cellular fluorescent detection and quantitative determination were then performed using a flow cytometer (Beckman Coulter, USA).

### 2.10. Determination of the Mitochondrial Transmembrane Potential

JC-1 was used to detect changes in the mitochondrial transmembrane potential. Cells were incubated with 10 *μ*L of 200 *μ*M JC-1 (final concentration, 2 *μ*M) for 30 min in the dark and analyzed using an SC500 flow cytometer.

### 2.11. Western Blotting

Equal amounts of protein from each sample cell were separated by SDS-PAGE and transferred to polyvinylidene difluoride membranes. The membranes were then blocked with 5% (*w*/*v*) nonfat milk powder for 2 h and incubated overnight with primary antibodies at 4°C. After washing, the membranes were incubated with horseradish peroxidase-conjugated secondary antibody for 1 h at room temperature. Finally, the protein bands were visualized using the ECL chemiluminescence detection system (Amersham, USA).

### 2.12. Statistical Analysis

Descriptive analyses were used to present the results of the chemical composition analysis and the antioxidant activity assays. The analysis of variance and Tukey's multiple range test were used to test the differences between groups, and a *P* value less than 0.05 was considered significant. The data were analyzed using SPSS 17.0 software (SPSS, Chicago, IL, USA).

## 3. Results

### 3.1. Identification of the Antioxidant Fraction

**Figure 1:**
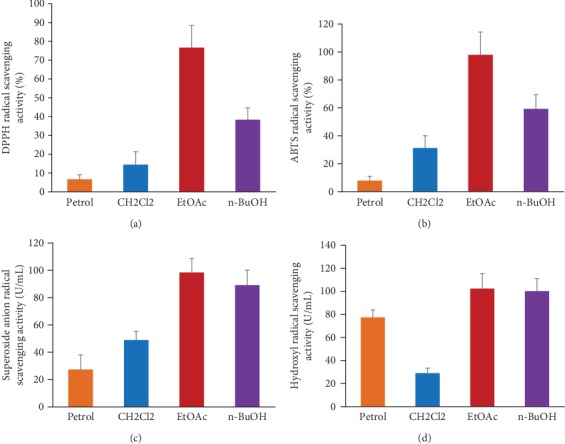
Antioxidant activity of the four obtained fractions of *D. moldavica L*. ethanol extract: (a) DPPH, (b) ABTS, (c) superoxide radical, and (d) hydroxyl radical.

Four *in vitro* antioxidant activity assays were performed to identify the fraction of *D. moldavica L*. ethanol extract that exhibits the antioxidant activity, including DPPH, ABTS, hydroxyl, and superoxide anion radical scavenging assays. As shown in Figure 1, the EtOAc fraction of *D. moldavica L*. ethanol extract possessed the highest scavenging ability, as demonstrated by the four assays. Moreover, the highest total flavonoid content was found in the EtOAc fraction, followed by the n-BuOH, CH_2_Cl_2_, and petrol fractions. Similarly, the total phenolic contents were ranked in the order of EtOAc>n-BuOH>CH2Cl2>petrol (Table 1). Additionally, the chromatograms also showed that rosmarinic acid, tilianin, luteolin, apigenin, and disometin were found in the EtOAc fraction (Figure S1).

### 3.2. The EtOAc Fraction of *D. moldavica L*. Ethanol Extract Protected H9c2 Cells against H_2_O_2_-Induced Cytotoxicity

**Figure 2:**
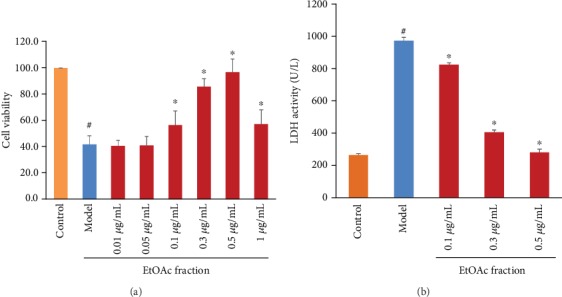
Effects of the EtOAc fraction of *D. moldavica L*. ethanol extract on cell viability and LDH activity in H_2_O_2_-treated H9c2 cells. (a) Cell viability. (b) LDH activity. Values are presented as the mean ± SD. ^∗^*P* < 0.05, *vs.* the model group. ^#^*P* < 0.05, *vs.* the control group.

To assess the effects of the EtOAc fraction of *D. moldavica L*. ethanol extract on H_2_O_2_-induced cytotoxicity in H9c2 cells, the cell viability was determined. As shown in Figure 2(a), the cell viability was significantly decreased in the H_2_O_2_-treated group compared with the control group. The EtOAc fraction at the doses of 0.1, 0.3, 0.5, and 1.0 *μ*g/mL significantly attenuated H_2_O_2_-induced reduction in cell viability, in comparison with the model group. The results further showed that cell viability was increased in a dose-dependent manner when the EtOAc fraction concentration was increased from 0.1 to 0.5 *μ*g/mL. However, compared with the model group, the EtOAc fraction had no significant effect on cell viability at the doses of 0.01 and 0.05 *μ*g/mL. Therefore, the doses of 0.1, 0.3, and 0.5 *μ*g/mL of the EtOAc fraction were selected for further experiments. Moreover, cytotoxicity was studied by determining the LDH activity. The LDH activity was remarkably high in the model group as compared to control. There were significant decreases in the LDH activity after increasing the doses of the EtOAc fraction (0.1, 0.3, and 0.5 *μ*g/mL) in comparison with the model group (Figure 2(b)).

### 3.3. The EtOAc Fraction of *D. moldavica L*. Ethanol Extract Inhibited H_2_O_2_-Induced Oxidative Damage in H9c2

**Figure 3:**
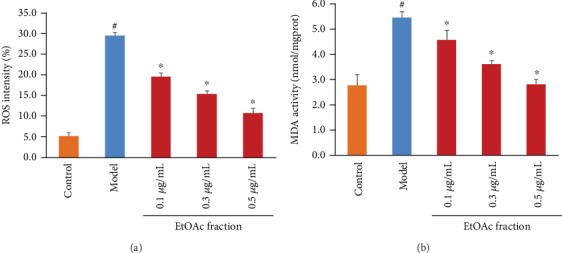
Effects of the EtOAc fraction of *D. moldavica L*. ethanol extract on ROS production and MDA level in H_2_O_2_-treated H9c2 cells. (a) ROS production. (b) MDA level. Values are presented as the mean ± SD. ^∗^*P* < 0.05, *vs.* the model group. ^#^*P* < 0.05, *vs.* the control group.

**Figure 4:**
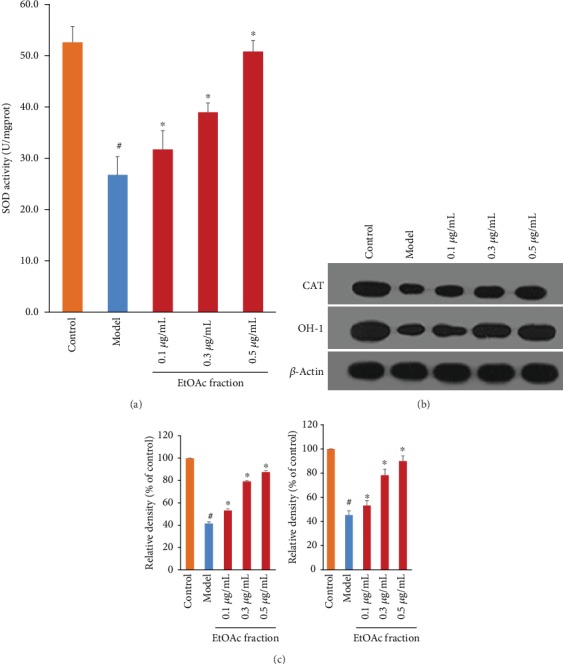
Effects of the EtOAc fraction of *D. moldavica L*. ethanol extract on the level of total SOD and the expression of CAT and HO-1 in H_2_O_2_-treated H9c2 cells. (a) The level of total SOD. (b) The expression of CAT and HO-1. (c) Densitometry analysis of CAT (left) and HO-1 (right) levels. Values are presented as the mean ± SD. ^∗^*P* < 0.05, *vs.* the model group. ^#^*P* < 0.05, *vs.* the control group.

To determine whether or not the EtOAc fraction can protect H9c2 cells in response to H_2_O_2_ injury, we examined H_2_O_2_-induced alterations in ROS production and MDA level in the presence of the EtOAc fraction in H9c2 cells. Our results showed that the EtOAc fraction treatment significantly reduced ROS production (Figure 3(a)). The EtOAc fraction treatment also significantly prevented the H_2_O_2_-induced MDA levels in a dose-dependent manner (Figure 3(b)). We further measured the effects of the EtOAc fraction on the expression of free radical scavenging enzymes as well as the level of total SOD. As shown in Figure 4, the EtOAc fraction treatment significantly increased the expression of CAT and HO-1 compared with the control group. Besides, the EtOAc fraction treatment restored the H_2_O_2_-induced decrease in the total SOD in a dose-dependent manner.

### 3.4. The EtOAc Fraction of *D. moldavica L*. Ethanol Extract Inhibited H_2_O_2_-Induced Mitochondrial Damage in H9c2

**Figure 5:**
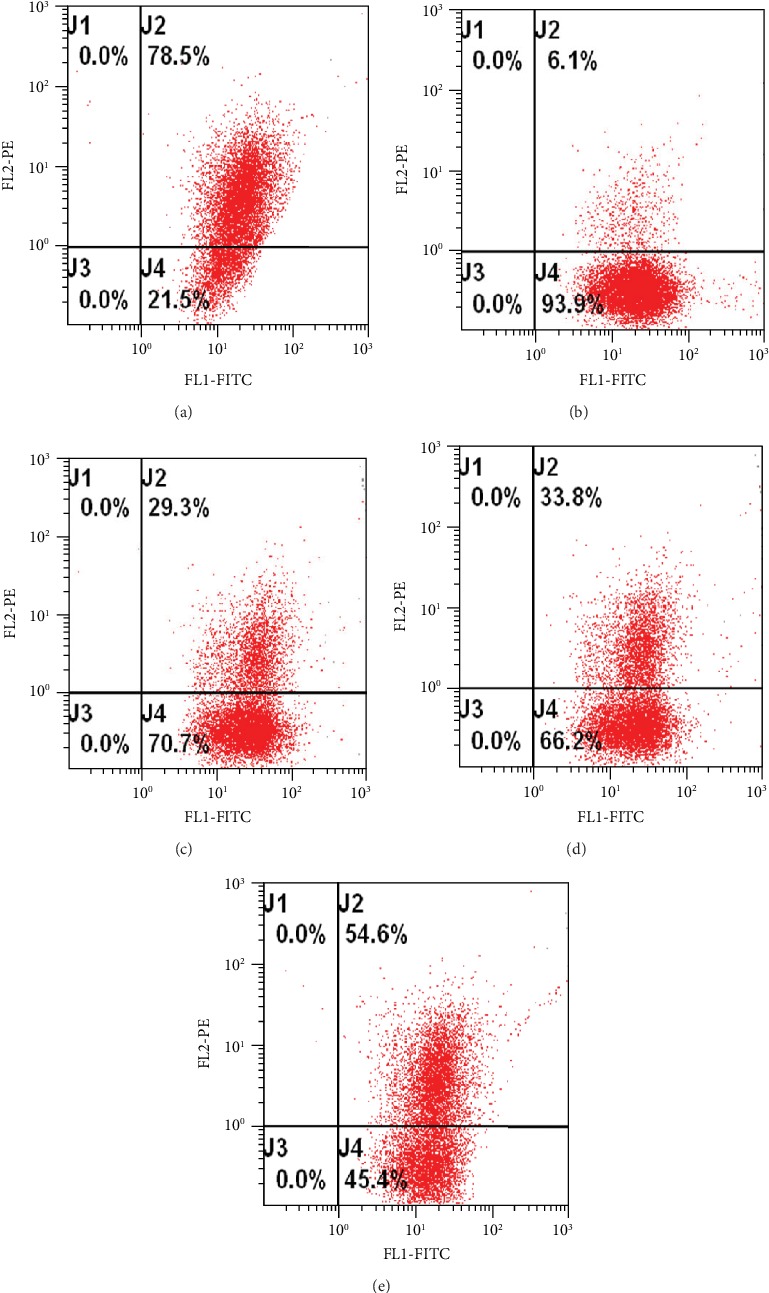
Effects of the EtOAc fraction of *D. moldavica L*. ethanol extract on the mitochondrial membrane potential in H_2_O_2_-treated H9c2 cells. (a) Control group. (b) Model group. (c) EtOAc fraction group (0.1 *μ*g/mL). (d) EtOAc fraction group (0.3 *μ*g/mL). (e) EtOAc fraction group (0.5 *μ*g/mL). Values are presented as the mean ± SD. ^∗^*P* < 0.05, *vs.* the model group. ^#^*P* < 0.05, *vs.* the control group.

To determine whether or not the EtOAc fraction can protect mitochondria in response to H_2_O_2_ injury, we examined H_2_O_2_-induced alterations in mitochondrial membrane potential in the presence of 0.5 *μ*g/mL EtOAc fraction in H9c2 cells using JC-1 dye. We found that the EtOAc fraction prevented the H_2_O_2_-induced impairment in mitochondrial membrane potential in a dose-dependent manner (Figure 5).

### 3.5. The EtOAc Fraction of *D. moldavica L*. Ethanol Extract Inhibited H_2_O_2_-Induced H9c2 Apoptosis

**Figure 6:**
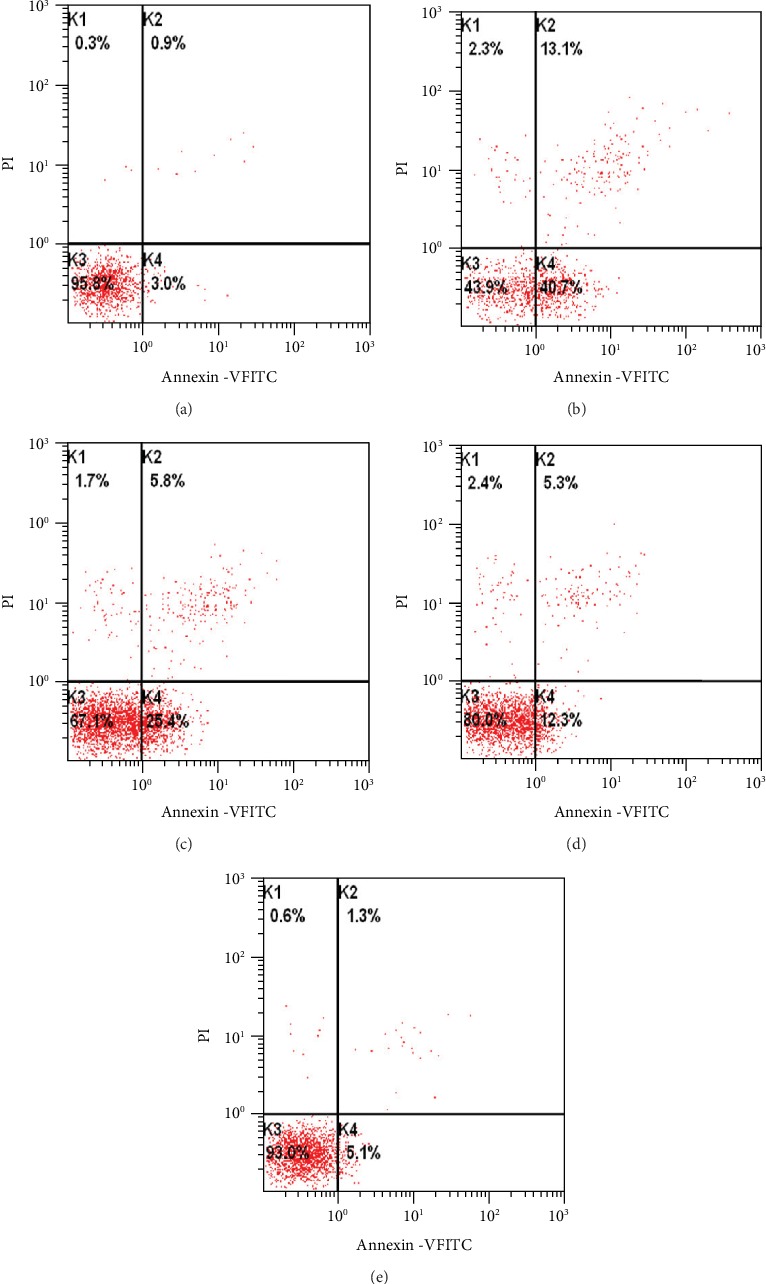
Effects of the EtOAc fraction of *D. moldavica L*. ethanol extract on apoptosis in H_2_O_2_-treated H9c2 cells. (a) Control group. (b) Model group. (c) EtOAc fraction group (0.1 *μ*g/mL). (d) EtOAc fraction group (0.3 *μ*g/mL). (e) EtOAc fraction group (0.5 *μ*g/mL). Values are presented as the mean ± SD. ^∗^*P* < 0.05, *vs.* the model group. ^#^*P* < 0.05, *vs.* the control group.

To examine the effects of the EtOAc fraction on H_2_O_2_-induced H9c2 apoptosis, the percentage of apoptotic cells was detected by flow cytometry. The apoptotic rate was increased in the H_2_O_2_ group, while the EtOAc fraction treatment decreased the apoptotic rate in a dose-dependent manner (Figure 6).

### 3.6. The EtOAc Fraction of *D. moldavica L*. Ethanol Extract Regulated the Apoptosis-Related Protein Expression in H9c2

**Figure 7:**
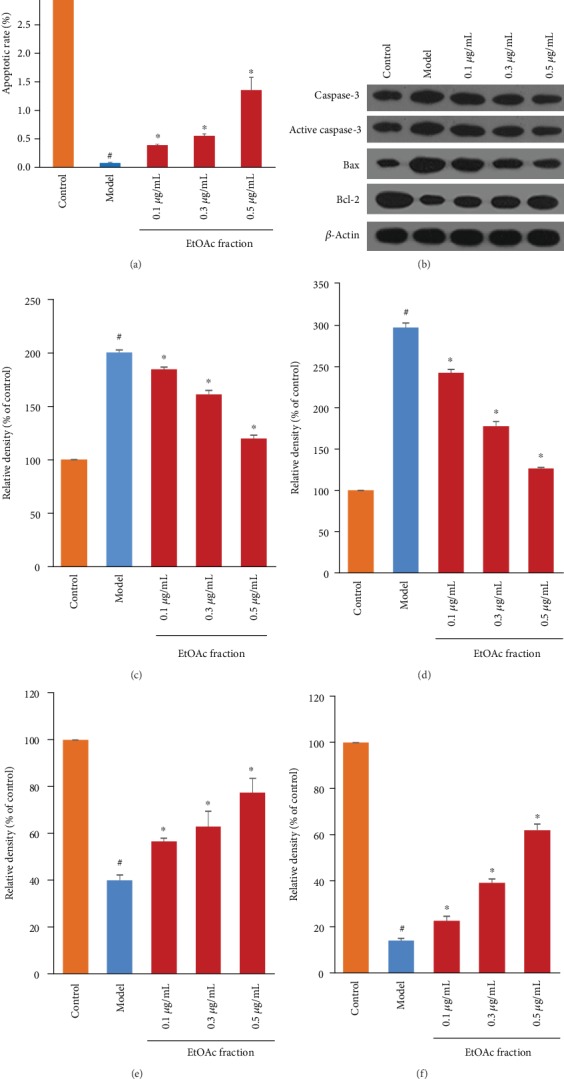
Effects of the EtOAc fraction of *D. moldavica L*. ethanol extract on the apoptotic rate and apoptosis-related protein expression in H_2_O_2_-treated H9c2 cells. (a) Effects of the EtOAc fraction of *D. moldavica L*. ethanol extract on the apoptotic rate. (b) Representative western blotting analysis of the protein expression levels of caspase-3, active caspase-3, Bax, and Bcl-2. (c–f) Densitometry analysis of caspase-3, active caspase-3, Bax, and Bcl-2 levels, respectively. Values are presented as the mean ± SD. ^∗^*P* < 0.05, *vs.* the model group. ^#^*P* < 0.05, *vs.* the control group.

The effects of the EtOAc fraction on apoptosis-related protein expression were also measured by western blotting. We found that the EtOAc fraction treatment significantly decreased the expression of the proapoptotic proteins caspase-3 and Bax, while it enforced the expression of antiapoptotic protein Bcl-2 in a dose-dependent manner when compared with the model group (Figure 7).

## 4. Discussion

*D. moldavica L*. is one of the most widely used medicinal herbs in traditional Chinese medicine. Previous studies demonstrated that the extracts of *D. moldavica L*. display antioxidant activities [19]. The myocardial protective function of total flavonoid extract from *D. moldavica L*. has also been reported [13, 20]. Recently, our group further demonstrated that *D. moldavica L*. attenuated cerebral ischemia-reperfusion injury in rats by inhibiting inflammation and oxidative stress [21]. To date, however, the exact molecular mechanisms of action of *D. moldavica L*. on responses to H_2_O_2_-induced injury in H9c2 cells have not been clearly defined. In the study, we identified the antioxidant fraction of *D. moldavica L*. ethanol extract and investigated its cardioprotective effects and possible mechanisms.

The preliminary antioxidant activity results showed that the EtOAc fraction of *D. moldavica L*. ethanol extract exhibited the remarkable highest antioxidant activity in the four obtained fractions. Additionally, our results revealed that the EtOAc fraction contained the highest abundance of flavonoids and phenols, which are shown to have strong antioxidant activities [22, 23]. As shown in Figure S1, we further found that tilianin and rosmarinic acid were the main compounds in the EtOAc fraction of *D. moldavica L*. ethanol extract. The presence of rosmarinic acid in *D. moldavica L*. has antioxidant potential and radical scavenging activity [24]. Tilianin from *D. moldavica L*. was proved to display anti-inflammatory activity [25]. Therefore, the antioxidant properties of the EtOAc fraction can be explained by its phytoconstituents, such as tilianin and rosmarinic acid, which were representative phenolic and flavonoid compounds of *D. moldavica L*. These results confirmed for the first time that the EtOAc fraction mediated the antioxidant properties of *D. moldavica L*.

In this study, we showed that the EtOAc fraction significantly increased the cell viability, reduced LDH release, inhibited ROS production, and decreased MDA level in H_2_O_2_-treated H9c2 cells. Additionally, the EtOAc fraction enhanced the SOD level and the expression of CAT and HO-1. It is well-known that these critical antioxidant enzymes play a major role in ROS scavenging [26]. As already mentioned, extracts of *D. moldavica L*. could reduce the MDA level and increase the contents of SOD and CAT in diabetic rats [27]. Our results were consistent with previous findings. Overall, the results confirmed that the EtOAc fraction prevents H9c2 cells against H_2_O_2_-induced oxidative stress.

It is reported that oxidative stress-induced excess intracellular ROS can damage the mitochondrial membrane potential and result in cell apoptosis [26]. In the present study, we evaluated the effect of the EtOAc fraction on the mitochondrial membrane potential of H9c2 cells exposed to oxidative stress. We found that the EtOAc fraction maintained the mitochondrial function and inhibited the apoptosis of H9c2 cells in response to oxidative stress. Previous studies also demonstrated that total flavonoid extract from *D. moldavica L*. attenuated ischemia-reperfusion-induced myocardial apoptosis [14], which agreed well with our results.

To further understand the effect of the EtOAc fraction on apoptosis, we measured the expression of apoptosis-related proteins by western blotting analysis. Our results showed that the EtOAc fraction can increase the expression of the antiapoptotic protein (Bcl-2) and inhibit the expression of proapoptotic proteins (Bax), which play crucial pathophysiological roles in cardiomyocyte apoptosis following H_2_O_2_ injury. The result is in agreement with previous studies [28]. This study further revealed that the EtOAc fraction decreased H_2_O_2_-induced caspase-3 activity, which was the most important apoptotic factor [29]. These results suggested that regulation of mitochondrial function and apoptosis partially contributed to the EtOAc fraction-induced cardioprotection against oxidative stress. Further studies might be required to investigate the effect of other pathways on H_2_O_2_-induced myocardial injury.

In conclusion, the present study provided, for the first time, evidence that the EtOAc fraction of *D. moldavica L*. ethanol extract exhibited significant antioxidant activity. Meanwhile, the study revealed that the EtOAc fraction protected H9c2 cardiomyocytes against H_2_O_2_-induced cytotoxicity and oxidative stress. The underlying mechanisms are possibly associated with the preservation of the mitochondrial function and attenuation of cardiomyocyte apoptosis. However, further studies are needed to investigate other pathways involved in the cardioprotective effect of the EtOAc fraction of *D. moldavica L*. ethanol extract. These findings provide a scientific basis for its ethnomedical use in the treatment of cardiovascular diseases.

## Figures and Tables

**Table 1:** Total phenolic content (TFC) and total flavonoid content (TPC) of different polar fractions of *D. moldavica L*. ethanol extract^a^.

Fractions	Yield (mg/g)	TFC (mg RE/g)	TPC (mg GAE/g)
Petrol	8.51 ± 0.09	7.65 ± 0.26	16.49 ± 0.24
CH_2_Cl_2_	8.62 ± 0.07	19.15 ± 0.24	142.45 ± 1.52
EtOAc	9.33 ± 0.06	65.04 ± 0.57	511.05 ± 1.91
n-BuOH	19.55 ± 0.21	50.13 ± 0.32	294.38 ± 1.54

^a^Values are presented as the mean ± SD.

## Data Availability

All data generated or analyzed during this study are included in this published article or available from the corresponding author upon reasonable request.

## Supplementary Materials

Supplementary Materials Figure S1: HPLC-DAD chromatograms of EtOAc fraction of D. moldavica L. ethanol extract, visualized at 330 nm. 1, rosmarinic acid; 2, tilianin; 3, luteolin; 4, an unknown compound; 5, apigenin; 6, disometin.
