# Supplementary material for: Dracocephalum moldavica L. Extracts Protect H9c2 Cardiomyocytes against H2O2-Induced Apoptosis and Oxidative Stress
Source: Biomed Res Int. 2020 May 2;2020:8379358. doi: 10.1155/2020/8379358 (PMC7222556; doi:10.1155/2020/8379358)
Supplement: Supplementary Materials — Figure S1: HPLC-DAD chromatograms of EtOAc fraction of D. moldavica L. ethanol extract, visualized at 330 nm. 1, rosmarinic acid; 2, tilianin; 3, luteolin; 4, an unknown compound; 5, apigenin; 6, disometin. [file 8379358.f1.pdf]

Supplementary Material

Dracocephalum moldavica L. extracts protect H9c2 cardiomyocytes against H_2O_2-induced apoptosis and oxidative stress

Min Jin[1, 2], Hui Yu[3], Xia Jin[4], Lailai Yan[1], Jingyu Wang[1]*, and Zhanli Wang[2, 3]*

[1] Center of Medical & Health Analysis, School of Public Health, Peking University, Beijing100191, China

[2] School of Public Health, Baotou Medical College, Baotou 014040, China

[3] Inner Mongolia Key Laboratory of Disease-Related Biomarkers, The Second Affiliated Hospital, Baotou Medical College, Baotou 014040, China

[4] Inner Mongolia Autonomous Region People's Hospital, Hohhot 010010, China

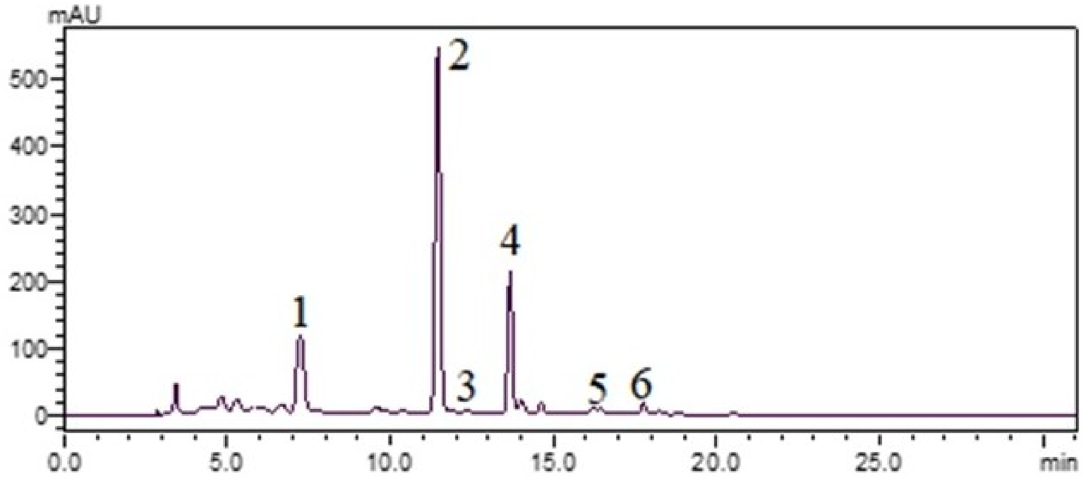

Figure S1. HPLC-DAD chromatograms of EtOAc fraction of *D. moldavica L.* ethanol extract, visualized at 330 nm. 1, rosmarinic acid; 2, tilianin; 3, luteolin; 4, an unknown compound; 5, apigenin; 6, disometin.
